# Chicago Dental Society

**Published:** 1889-06

**Authors:** 


### Chicago Dental Society.

At the annual meeting of the Chicago Dental Society held on Tuesday evening April 2, 1889, the following named were elected officers for the ensuing year : P. J. Kester, President; D. M. Cattell, First Vice-President; W. J. Martin, Second Vice-President; A. E. Baldwin, Secretary; Louis Ottofy, Cor. Secretary; E. D. Swain, Treasurer; A. W. Harlan, Librarian. Member of the Executive Committee, J. Austin Dunn; Board of Censors, F. H. Gardiner, C. F. Hartt, and S. S. Davis. Delegates to the International Dental Congress at Paris, France, September 1 to 8, 1889, were appointed as follows: A. W. Harlan (Secretary), J. M. Crouse, T. W. Brophy, J. A. Swasey, P. J. Kester, W. W. Allport, A. E. Baldwin, Louis Ottofy, S. S. Davis, J. W. Wassall, and W. B. Ames.

A report from Dr. Crouse showed the Dental Protective Association of the United States to be flourishing, and dentists throughout the United States are requested to become members of this association. LOUIS OTTOFY, Cor. Sec.

THE TREATMENT OF TETANUS.—Tetanus may be considered to be an infectious disease dependent on bacillary infection. The symptoms are due, not to the irritation caused by the presence of the bacilli, but to poisonous effects of the products elaborated by them. In the earlier stages the bacilli do not spread beyond the site of infection, and the first indication for treatment is to destroy or remove the infected tissues. This can be done by means of free incisions, scraping, and the application of nitric or sulphuric acid. The next step is to facilitate the elimination of the toxic products, and this object will be attained by stimulating the secretion, as by purgatives, diuretics, and diaphoretics, jaborandi having been found peculiarly useful for this very reason. Lastly, the irritating effects of the poison on the various systems must be controlled by sedatives, preferably chloral hydrate. Several cases are already on record in which a cure has been effected, and we have at any rate a rational and scientific method of treatment for what was hitherto considered to be a hopeless malady. —*Med. Press and Circular.*

